# Vestibular Evoked Myogenic Potentials (cVEMP and oVEMP) in Pregnancy: A Clinical Study

**DOI:** 10.3390/audiolres16030080

**Published:** 2026-05-26

**Authors:** Isil Cakmak Karaer, Erdinc Sarıdogan

**Affiliations:** 1 Department of Otolaryngology, School of Medicine, Turgut Ozal University, Battalgazi, Malatya 44901, Türkiye; 2 Department of Otolaryngology, Malatya Training and Research Hospital, Hastane Street No: 44, Battalgazi, Malatya 44623, Türkiye; 3 Department of Obstetrics and Gynecology, School of Medicine, Inonu University, Battalgazi, Malatya 44280, Türkiye; erdinc_saridogan@hotmail.com

**Keywords:** pregnancy, vestibular evoked myogenic potentials, cVEMP, oVEMP, otolith organs, saccule, utricle

## Abstract

**Background/Objectives**: Pregnancy involves significant hormonal, cardiovascular, and physiological shifts that may potentially affect the vestibular system. The utricle and saccule, the two primary otolith organs, are responsible for detecting linear acceleration and maintaining equilibrium. This study aims to objectively evaluate the functional status of these organs throughout the three trimesters of pregnancy using Vestibular Evoked Myogenic Potentials (VEMPs). **Methods**: A prospective cross-sectional study was conducted with 64 healthy primigravida women (mean age 29.4 ± 2.3 years). Cervical VEMP (cVEMP) and ocular VEMP (oVEMP) tests were performed at three distinct time points: the first (8–9 weeks), second (20–21 weeks), and third (33–34 weeks) trimesters. Latencies (p13 and n23 for cVEMP; n10 and p15 for oVEMP) and peak-to-peak amplitudes were recorded and statistically compared across trimesters. **Results**: No statistically significant differences were found in cVEMP p13 and n23 latencies or amplitudes across the three trimesters (*p* > 0.05). Similarly, oVEMP p15 latencies and amplitudes remained stable throughout the pregnancy (*p* = 0.43 and *p* = 0.95, respectively). While a slight numerical increase in certain latencies was observed in the third trimester, and the oVEMP n10 latency was found to be consistently prolonged compared to non-pregnant norms, these values remained stable between trimesters. The inter-aural asymmetry showed no significant deviations, indicating a balanced vestibular adaptation. **Conclusions**: The functional integrity of the saccule and utricle is preserved during a healthy pregnancy. Despite the dramatic increase in gestational hormones such as estrogen and progesterone, the otolith-dependent reflex pathways (vestibulocollic and vestibulo-ocular) remain resilient. These results provide a clinical baseline for evaluating vestibular symptoms in pregnant populations, suggesting that significant VEMP abnormalities should prompt investigation into underlying vestibular pathologies rather than being attributed to normal pregnancy changes.

## 1. Introduction

The utricle and saccule are the specialized otolith organs of the inner ear’s vestibular system, primarily responsible for detecting linear acceleration and head orientation relative to gravity. By processing these mechanical stimuli, they play a fundamental role in maintaining postural stability, gaze control, and spatial orientation [1,2,3,4]. While the utricle is predominantly sensitive to horizontal linear acceleration and head tilt, the saccule detects vertical acceleration and gravitational changes [3,5]. Within both organs, specialized hair cells in the macula are covered by a gelatinous membrane embedded with calcium carbonate crystals (otoconia). Head movement triggers the displacement of these otoconia, subsequently deflecting the hair cells and generating neural signals that are integrated centrally for balance and autonomic regulation [1,2,6,7,8].

In clinical practice, the functional integrity of these organs is selectively evaluated through Vestibular Evoked Myogenic Potentials (VEMPs). These tests leverage the distinct neural projections from the otolith organs to the ocular and cervical musculature [4,9]. According to the American Academy of Neurology, the cervical VEMP (cVEMP) primarily assesses saccular function via the vestibulocollic reflex, whereas the ocular VEMP (oVEMP) evaluates utricular function through the vestibulo-ocular reflex [10]. Any dysfunction within these specialized pathways can manifest as vertigo, imbalance, or subclinical spatial disorientation.

Pregnancy involves hormonal, hemodynamic, and metabolic changes that may influence vestibular function. The physiological landscape of pregnancy is characterized by significant fluctuations in hormones—most notably estrogen and progesterone—which have been shown to modulate the vestibular system both directly and indirectly. Estrogen and progesterone receptors are widely expressed throughout the vestibular sensory network, suggesting that gestational hormonal shifts may alter inner ear homeostasis [11,12]. Elevated levels of these hormones, alongside systemic changes such as increased fluid retention, are associated with reversible alterations in vestibular function, often manifesting as subclinical dysfunction or contributing to pregnancy-induced dizziness and nausea [13,14,15].

Despite the known impact of pregnancy on the vestibular system, the specific effects on otolith organ function remain an area that requires objective quantification. This targeted approach allows for a clearer interpretation of whether the peripheral vestibular end-organs themselves are directly influenced by pregnancy-related physiological changes, independent of central or semi-circular canal involvement. Therefore, the aim of this study is to investigate the physiological changes occurring in the utricle and saccule during pregnancy by utilizing cVEMP and oVEMP parameters.

## 2. Materials and Methods

This prospective cross-sectional study was conducted at the Department of Otolaryngology, Malatya State Hospital, Türkiye. The study protocol was approved by the Malatya Ethical Review Board (Decision No: 2021-94) and performed in accordance with the ethical principles of the Declaration of Helsinki. All participants provided informed written consent prior to their inclusion in the study.

### 2.1. Study Participants

The study included 64 primigravida (first-time pregnant) women with a mean age of 29.4 ± 2.3 years.

Inclusion criteria were pregnant women aged 18–40 years with singleton pregnancies and no vestibular or neurotological disorders. Participants were excluded if they had systemic diseases, including diabetes mellitus, hypertension, hypothyroidism, or hyperthyroidism. We also excluded individuals regularly using medication during pregnancy or those with a history of vestibular and auditory disorders such as hyperemesis gravidarum, superior semicircular canal dehiscence (SSCD), Meniere’s disease, motion sickness, vertigo attacks, or any prior otological/neuro-otological surgery.

Vestibular evaluations were performed across three standardized time points: first (8–9 weeks), second (20–21 weeks), and third (33–34 weeks) trimesters.

### 2.2. VEMPs Procedures and Instrumentation

VEMP recordings were performed using the Eclipse EP25 Evoked Potential System (Interacoustics, Middelfart, Denmark). To ensure optimal recording quality, the subjects’ skin was thoroughly cleansed to maintain skin impedance below 10 kΩ. The recording protocols were adapted from established neurophysiological standards [16].

For both cVEMP and oVEMP, 500 Hz short tone bursts were utilized as stimuli, presented at a rate of 5.1 Hz with a 2-2-2 ms (rise/fall/plateau) envelope. A total of 200 stimuli were averaged per trial, and three trials (including one control trial) were obtained for each ear to ensure reproducibility. The analysis window was set to 100 ms, including a 20 ms pre-stimulation interval. A VEMP response was defined as “absent” if no identifiable, reproducible biphasic waveform was observed.

### 2.3. cVEMP Recording

The active electrode was placed on the upper third of the sternocleidomastoid (SCM) muscle, the ground electrode on the upper forehead (nasion), and the reference electrode on the jugular notch. During testing, patients sat upright with their heads rotated away from the stimulated side to achieve sufficient SCM contraction.

**Figure 1:**
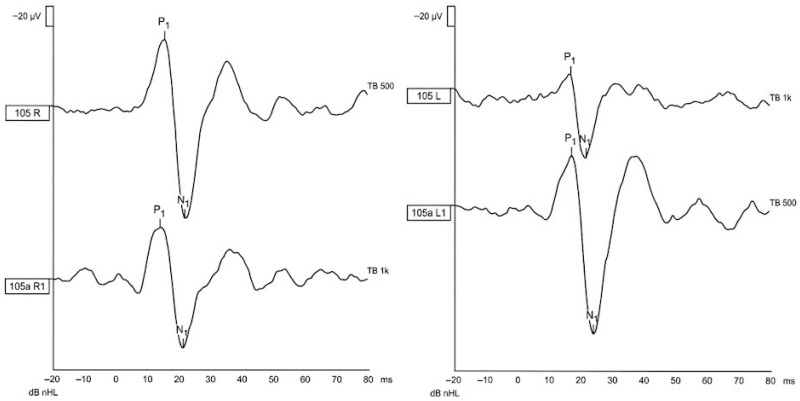
Representative cVEMP waveforms recorded from the right and left ears in response to 500 Hz and 1 kHz tone-burst stimuli. P1 and N1 peaks are indicated.

Real-time electromyographic (EMG) feedback was monitored to ensure a consistent tonic contraction of the SCM between 50 and 150 μV. Air-conducted rarefaction stimulation was delivered via 3M™ E-A-RLINK™ Insert Eartips (3M Company, St. Paul, MN, USA); two trials were performed at 100 dB nHL to confirm the presence of the response, and one trial at 80 dB nHL served as a control. Measured parameters included the positive (p13) and negative (n23) peak latencies (ms) and the peak-to-peak amplitude (μV) (Figure 1). Inter-aural symmetry was evaluated using the Asymmetry Ratio (AR), calculated with the formula: AR = 100 × (ARight − ALeft)/(ARight + ALeft), where ‘A’ represents the peak-to-peak amplitude.

### 2.4. oVEMP Recording

**Figure 2:**
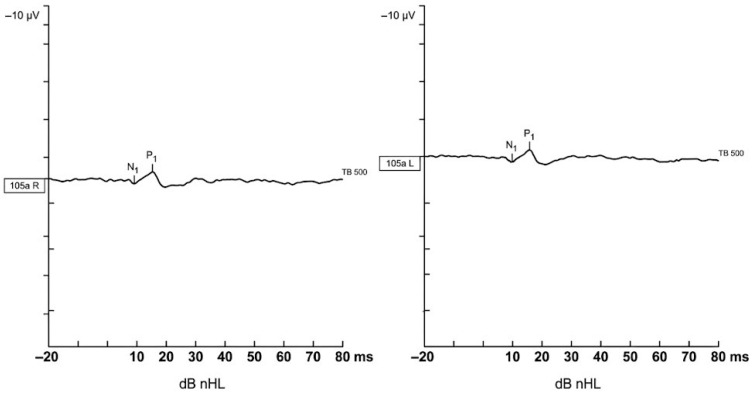
Representative oVEMP waveforms recorded from the right and left ears in response to 500 Hz tone-burst stimuli. N1 and P1 peaks are indicated.

Active electrodes were placed approximately 0.5 cm inferior to each eye, aligned with the lateral half of the lower eyelid [17]. The reference electrode was placed on the vertex, and the ground electrode remained on the lower forehead. Participants were instructed to maintain a maximal upward gaze (approximately 30°) during stimulation to activate the inferior oblique muscle. The recorded parameters consisted of the first negative peak (n10) and the subsequent positive peak (p15) latencies (ms), along with the peak-to-peak amplitudes (μV) (Figure 2). As with cVEMP, oVEMP inter-aural asymmetry was calculated and analyzed across all three trimesters.

### 2.5. Statistical Analysis

All statistical analyses were performed using SPSS 15.0 (SPSS^®^ for Windows 15.0, Chicago, IL, USA). The distribution of continuous variables was assessed using the Shapiro–Wilk test. Descriptive statistics were expressed as mean ± standard deviation (SD).

Since measurements were obtained from the same participants across three trimesters, repeated measures analysis was applied. For normally distributed variables, which included cVEMP latency, amplitude, AR, and oVEMP p15 latency and amplitude, one-way repeated measures ANOVA was utilized, with Mauchly’s test used to evaluate the sphericity assumption; when violated, the Greenhouse–Geisser correction was applied. Results are reported using the F-statistic for ANOVA and the Chi-square value for the Friedman test. A *p*-value < 0.05 was considered statistically significant.

## 3. Results

A total of 64 primigravida patients were enrolled in the study, with VEMP recordings successfully obtained across all three trimesters.

### 3.1. cVEMP Outcomes

Results of cVEMP are shown in Table 1. The cVEMP parameters, representing saccular integrity, remained largely consistent throughout the pregnancy. The mean values for p13 and n23 showed a slight numerical increase toward the third trimester; however, these changes did not reach statistical significance. The p13 was 14.9 ± 2.1 ms in the first trimester, 15.1 ± 5.4 ms in the second, and 18.8 ± 4.6 ms in the third (χ2(2) = 4.82, *p* = 0.09). Similarly, n23 were recorded as 9.8 ± 2.6 ms, 10.8 ± 5.7 ms, and 15.07 ± 4.2 ms across the trimesters, respectively (χ2(2) = 5.63, *p* = 0.06).

The mean amplitude values remained stable: 55.9 ± 40 μV in the first trimester, 48 ± 42 μV in the second trimester, and 58.3 ± 45 μV in the third trimester (F(2,126) = 0.56, *p* = 0.57). Notably, latency was measured as 5.1 ± 1 ms in the first trimester, 4.2 ± 1.1 ms in the second, and 3.7 ± 0.95 ms in the third. AR values were 0.26 ± 0.16 in the first trimester, 0.29 ± 0.13 in the second trimester, and 0.19 ± 0.23 in the third trimester (F(2,126) = 3.15, *p* = 0.052). No statistically significant difference was found in latency, amplitude and the AR values between the three trimesters (F(2,126) = 1.66, *p* = 0.2).

Data are presented as mean ± standard deviation (SD). A *p*-value < 0.05 was considered statistically significant. p13 latency and n23 latency represent the peak latencies of the biphasic waveform. Interpeak latency (p13–n23) was calculated as the time interval between p13 and n23 peaks.

Amplitude was defined as the peak-to-peak amplitude between p13 and n23.

AR = (LA − RA)/(LA + RA),

### 3.2. oVEMP Outcomes

Results of oVEMP are shown in Table 2. The oVEMP parameters, representing utricular integrity, remained largely consistent throughout the pregnancy. The p15 was 24.1 ± 2.4 ms in the first trimester, 22.7 ± 2.1 ms in the second, and 25.2 ± 7.8 ms in the third (F(2,126) = 1.86, *p* = 0.18). Similarly, n10 was recorded as 16.7 ± 2.5 ms, 17.2 ± 2.8 ms, and 18.2 ± 7.4 ms across the trimesters (χ2(2) = 1.69, *p* = 0.43).

The mean oVEMP amplitude was 9.6 ± 5.4 μV in the first trimester and 9.6 ± 6.3 μV in the second trimester, and it remained nearly unchanged in the third trimester at 9.9 ± 9.7 μV (F(2,126) = 0.01, *p* = 0.95). Notably, latency was measured as 7.3 ± 2.5 ms in the first trimester, 5.5 ± 2.0 ms in the second, and 7.1 ± 2.1 ms in the third (F(2,126) = 1.75, *p* = 0.2). AR value was 0.29 ± 0.25 in the first trimester, 0.31 ± 0.21 in the second trimester, and 0.32 ± 0.24 in the third trimester (χ2(2) = 0.28, *p* = 0.87).

Data are presented as mean ± standard deviation (SD). A *p*-value < 0.05 was considered statistically significant. p15 latency and n10 latency represent the peak latencies of the biphasic waveform. Interpeak latency (p15–n10) was calculated as the time interval between p15 and n10 peaks.

Amplitude was defined as the peak-to-peak amplitude between p15 and n10.

AR = (LA − RA)/(LA + RA),

## 4. Discussion

This study evaluated the functional integrity of the otolith organs—specifically the saccule, utricle, and the associated inferior and superior vestibular nerves—throughout the three trimesters of pregnancy using cVEMP and oVEMP testing. Our results indicate that despite the profound hormonal, cardiovascular, and physiological shifts experienced during pregnancy, there are no statistically significant changes in these vestibular parameters. While these may be characterized as “negative findings,” they carry substantial clinical weight by establishing a normative longitudinal baseline for a population frequently affected by vestibular symptoms.

The influence of pregnancy hormones, particularly estrogen and progesterone, on the vestibular system is well-documented in terms of symptomatology. Progesterone metabolites, such as allopregnanolone sulfate, have been linked to increased nausea and vomiting through their effects on brainstem regions and vestibular pathways [18]. Furthermore, estrogen modulates neurotransmitter function within these circuits, potentially increasing susceptibility to vestibular symptoms [11,12]. However, our data may indicate that in healthy primigravida women, these hormonal fluctuations do not translate into objective peripheral dysfunction.

Regarding cVEMP analysis, p13 and n23 latencies remained within clinically accepted ranges across all trimesters. Although we observed slight numerical increases toward the third trimester, these did not reach statistical significance. Rather than indicating a progressive decline, these stable values reinforce the reliability of cVEMP as a diagnostic tool during pregnancy. Similarly, for oVEMP, the consistent prolongation of n10 latency compared to non-pregnant norms—without progression across trimesters—may indicate a stable physiological adaptation rather than an emerging pathology.

Regarding oVEMP, which assesses utricular function via the superior vestibular nerve and the vestibulo-ocular reflex [10,16], we observed that n10 latencies remained within normal limits (12–18 ms). However, the p15 latencies (24.1 ± 2.4, 22.7 ± 2.1, and 25.2 ± 7.8 ms) were found to be above the traditional normal limit of 8–12 ms. Interestingly, this prolongation was observed consistently from the first trimester through the third. The isolated prolongation of these values, while amplitudes remained robust and stable (9.6–9.9 µV), suggests that while the reflex pathway is functionally intact, there may be a baseline slowing of signal transmission within the utricle–superior vestibular nerve–brainstem axis in the pregnant population compared to non-pregnant norms. Critically, since no significant difference was found between the trimesters, this latency shift likely represents a stable physiological state inherent to pregnancy rather than a progressive pathological decline. This finding is of high clinical importance: it may indicate that while “normal” values for pregnant women may differ slightly from the general population, the peripheral vestibular end-organs maintain functional resilience against the systemic and hormonal shifts in gestation.

Recent literature has increasingly emphasized the functional relevance of vestibular pathways beyond mere electrophysiological measurements. For instance, abnormalities in the vestibulo-ocular reflex (VOR) have been shown to correlate strongly with balance and gait impairment in patients with neurological conditions, highlighting the clinical importance of subtle vestibular dysfunction even in the absence of overt symptoms [19]. In addition, contemporary diagnostic approaches to bilateral vestibulopathy advocate for a more comprehensive evaluation that includes not only semicircular canal function but also otolith organ assessment through cVEMP and oVEMP testing, underlining the critical role of these pathways in vestibular integrity [20].

In this context, the absence of significant alterations in cVEMP and oVEMP parameters observed in our study may indicate that, unlike pathological conditions, normal pregnancy does not lead to measurable impairment in otolith-mediated reflex pathways. This finding supports the concept that physiological hormonal fluctuations, although capable of inducing subjective vestibular symptoms, do not necessarily translate into objective dysfunction at the level of the peripheral vestibular system.

Furthermore, emerging evidence indicates that sensory systems, including auditory and vestibular pathways, can influence broader functional outcomes such as physical activity and mobility [21]. Although such relationships have not been directly investigated in pregnant populations, our findings provide a foundation for future studies exploring whether subtle vestibular changes—if present—may have functional implications in balance, gait, or fall risk during pregnancy. By “ruling out” peripheral otolith dysfunction as a standard consequence of healthy gestation, this study narrows the diagnostic focus for pregnancy-related balance disorders toward central and multisensory integration mechanisms.

Furthermore, emerging evidence indicates that sensory systems, including auditory and vestibular pathways, can influence broader functional outcomes such as physical activity and mobility [21]. Although such relationships have not been directly investigated in pregnant populations, our findings provide a foundation for future studies exploring whether subtle vestibular changes—if present—may have functional implications in balance, gait, or fall risk during pregnancy.

One of the primary clinical implications of this study is the validation of VEMP testing as a robust diagnostic instrument in the pregnant population. Because healthy pregnancy does not inherently impair otolith organ function, any significant VEMP abnormality or inter-aural asymmetry found in a pregnant patient should be treated as a potential indicator of an underlying vestibular disorder—such as Meniere’s disease or vestibular neuritis—rather than being dismissed as a normal consequence of gestation.

The focus of this study was limited to otolith-mediated peripheral reflex pathways. While we have demonstrated peripheral stability, the subjective dizziness often reported by pregnant women may instead arise from changes in central vestibular processing, multisensory integration, or hemodynamic shifts. Future research should explore how the brain integrates vestibular signals with changing proprioceptive and visual inputs as maternal body morphology and center of gravity shift. Investigating these central correlates will be essential to provide a comprehensive understanding of pregnancy-related balance disorders.

## 5. Conclusions

In conclusion, this study demonstrates that a healthy pregnancy does not significantly impair the functional capacity of the saccule and utricle. By providing a longitudinal normative baseline, these results offer a crucial reference point for clinicians. The overall stability of VEMP amplitudes and symmetry may indicate that the peripheral vestibular system remains resilient to gestational changes. Consequently, VEMP testing remains a reliable diagnostic tool during pregnancy, and clinicians should investigate significant abnormalities as markers of underlying pathology rather than physiological changes.

## Figures and Tables

**Table 1:** cVEMP parameters across pregnancy trimesters.

Parameter	1st Trimester	2nd Trimester	3rd Trimester	*p*-Value
p13 latency (ms)	14.9 ± 2.1	15.1 ± 5.4	18.8 ± 4.6	0.09
n23 latency (ms)	9.8 ± 2.6	10.8 ± 5.7	15.07 ± 4.2	0.06
Interpeak latency (p13–n23) (ms)	5.1 ± 1.0	4.2 ± 1.1	3.7 ± 0.95	0.052
Amplitude (µV)	55.9 ± 40	48 ± 42	58.3 ± 45	0.57
Asymmetry ratio	0.26 ± 0.16	0.29 ± 0.13	0.19 ± 0.23	0.20

**Table 2:** oVEMP parameters across pregnancy trimesters.

Parameter	1st Trimester	2nd Trimester	3rd Trimester	*p*-Value
P15 latency (ms)	24.1 ± 2.4	22.7 ± 2.1	25.2 ± 7.8	0.18
N10 latency (ms)	16.7 ± 2.5	17.2 ± 2.8	18.2 ± 7.4	0.43
Interpeak latency (n10–p15) (ms)	7.3 ± 2.5	5.5 ± 2.0	7.1 ± 2.1	0.20
Amplitude (µV)	9.6 ± 5.4	9.6 ± 6.3	9.9 ± 9.7	0.95
Asymmetry ratio	0.29 ± 0.25	0.31 ± 0.21	0.32 ± 0.24	0.87

## Data Availability

The datasets generated and analyzed during the current study are not publicly available due to privacy and ethical restrictions but are available from the first author, Dr. Isıl Cakmak Karaer, upon reasonable request.
